# Benefits from Below: Silicon Supplementation Maintains Legume Productivity under Predicted Climate Change Scenarios

**DOI:** 10.3389/fpls.2018.00202

**Published:** 2018-02-20

**Authors:** Scott N. Johnson, James M. W. Ryalls, Andrew N. Gherlenda, Adam Frew, Susan E. Hartley

**Affiliations:** ^1^Hawkesbury Institute for the Environment, Western Sydney University, Penrith, NSW, Australia; ^2^York Environmental Sustainability Institute, Department of Biology, University of York, York, United Kingdom

**Keywords:** alfalfa, aphids, atmospheric change, climate change, global warming, silica, silicon

## Abstract

Many studies demonstrate that elevated atmospheric carbon dioxide concentrations (eCO_2_) can promote root nodulation and biological nitrogen fixation (BNF) in legumes such as lucerne (*Medicago sativa*). But when elevated temperature (eT) conditions are applied in tandem with eCO_2_, a more realistic scenario for future climate change, the positive effects of eCO_2_ on nodulation and BNF in *M. sativa* are often much reduced. Silicon (Si) supplementation of *M. sativa* has also been reported to promote root nodulation and BNF, so could potentially restore the positive effects of eCO_2_ under eT. Increased nitrogen availability, however, could also increase host suitability for aphid pests, potentially negating any benefit. We applied eCO_2_ (+240 ppm) and eT (+4°C), separately and in combination, to *M. sativa* growing in Si supplemented (Si+) and un-supplemented soil (Si-) to determine whether Si moderated the effects of eCO_2_ and eT. Plants were either inoculated with the aphid *Acyrthosiphon pisum* or insect-free. In Si- soils, eCO_2_ stimulated plant growth by 67% and nodulation by 42%, respectively, whereas eT reduced these parameters by 26 and 48%, respectively. Aphids broadly mirrored these effects on Si- plants, increasing colonization rates under eCO_2_ and performing much worse (reduced abundance and colonization) under eT when compared to ambient conditions, confirming our hypothesized link between root nodulation, plant growth, and pest performance. Examined across all CO_2_ and temperature regimes, Si supplementation promoted plant growth (+93%), and root nodulation (+50%). *A. pisum* abundance declined sharply under eT conditions and was largely unaffected by Si supplementation. In conclusion, supplementing *M. sativa* with Si had consistent positive effects on plant growth and nodulation under different CO_2_ and temperature scenarios. These findings offer potential for using Si supplementation to maintain legume productivity under predicted climate change scenarios without making legumes more susceptible to insect pests.

## Introduction

Projected increases in atmospheric carbon dioxide (CO_2_) have been shown experimentally to stimulate biological nitrogen fixation (BNF) in legumes (Soussana and Hartwig, 1996; Zanetti et al., 1996; Hungate et al., 1999; Edwards et al., 2006; Lam et al., 2012). These effects are strongest immediately after exposure to elevated CO_2_ (eCO_2_) (Hungate et al., 2004) and when other nutrients (especially phosphorus) are not limiting (Rogers et al., 2009). Elevated CO_2_ (eCO_2_) can promote BNF via several mechanisms, including larger numbers of N_2_ fixing symbiotic bacteria in the rhizosphere (Schortemeyer et al., 1996), increased numbers of nodules which house N_2_ fixing rhizobia bacteria (Ryle and Powell, 1992) and enhanced nitrogenase activity (Norby, 1987). Broadly speaking, eCO_2_ allows legumes to increase rates of photosynthesis and allocate more carbon belowground to support increased root nodulation and therefore BNF (Aranjuelo et al., 2014).

Researchers are becoming increasingly aware of the importance of testing multiple environmental change factors because they are predicted to occur concurrently and often have either synergistic or antagonistic impacts on one another (Robinson et al., 2012; Johnson and Jones, 2017). Climate models predict, for instance, that air temperatures will increase in tandem with increases in atmospheric CO_2_ and warmer temperature may negate any positive impacts of eCO_2_ on plant growth (Newman et al., 2011). This may be particularly true in legume systems because higher temperatures can have inhibitory effects on BNF due to the relatively low tolerance of N_2_-fixing bacteria to higher temperatures (Zahran, 1999; Whittington et al., 2013; Aranjuelo et al., 2014). The optimal temperature for root nodule symbiosis for temperate legumes is thought to be around 15–25°C, above which detrimental effects can become evident (Aranjuelo et al., 2014). Elevated temperature (eT) can directly hinder the development and functionality of root nodulation and accelerate nodule senescence (Piha and Munns, 1987; Aranjuelo et al., 2006). In addition, eT can inhibit nodulation via plant-mediated mechanisms, including reduced root hair formation, fewer nodulation sites and poorer adherence of bacteria to root hairs (Hungria and Franco, 1993; Hungria and Vargas, 2000; Aranjuelo et al., 2014).

Soil conditions play an important role in determining the extent to which eCO_2_ and eT affect root nodulation in legumes (Aranjuelo et al., 2014). Several studies report that supplementation of soil silicon (Si) levels promotes growth in legumes (Horst and Marschner, 1978; Miyake and Takahashi, 1985; Guo et al., 2006; Johnson et al., 2017), though we know less about the functional role of Si in legumes compared to other plant families such as the Poaceae (Epstein, 1999; Cooke and Leishman, 2011). Moreover, Si supplementation can increase rates of root nodulation and symbiosis with nitrogen fixing bacteria (Nelwamondo and Dakora, 1999; Mali and Aery, 2008). However, how these positive effects of Si on nodulation are affected by eCO_2_ or eT, alone or in combination, have not yet been addressed. If Si could maintain nodulation rates under future climate change scenarios, such as eT, which usually decrease it, then such supplementation could be important in the mitigation of climate change impacts on agriculture.

While rhizobial colonization promotes legume growth and vigor, this improved host quality can also increase susceptibility to belowground (Quinn and Hower, 1986; Gerard, 2001; Johnson and McNicol, 2010) and aboveground insect herbivores (Dean et al., 2009, 2014; Kempel et al., 2009; Katayama et al., 2010; Whitaker et al., 2014). Beneficial effects of rhizobia on herbivores most likely arise through increased provision of nitrogen, which is frequently limiting in insect herbivore diets (Mattson, 1980). Increased provision of nitrogen may, however, allow plants to invest in plant defenses with negative impacts on herbivores (Pineda et al., 2010; Brunner et al., 2015). While Si supplementation usually increases plant resistance to herbivores (mainly reported in the Poaecae; Reynolds et al., 2009), it may also indirectly increase susceptibility to herbivores via increases in legume growth and nutritional quality (Johnson et al., 2017).

The objective of this study was to determine how eCO_2_ and eT, acting alone and in combination, affected root nodulation and plant growth in *Medicago sativa* in untreated (Si-) and Si supplemented (Si+) soil. We additionally aimed to establish whether these factors affected the abundance and colonization success of an insect herbivore (the aphid *Acyrthosiphon pisum*). We hypothesized that eCO_2_ increases growth and root nodulation in *M. sativa* but eT negates these effects. Si supplementation increases nodulation, even under eT, and therefore maximizes plant growth regardless of CO_2_ and temperature conditions. We hypothesized that aphid abundance would be positively linked to plant growth and nodulation, whether driven by Si supplementation or changes in CO_2_ and temperature conditions.

## Materials and Methods

### Insect Cultures and Plant Material

Four *A. pisum* cultures were established from a single parthenogenetic adult female collected from a pasture containing grasses and legumes, including lucerne, at the Hawkesbury Campus of the Western Sydney University, Penrith, NSW, Australia (latitude -33.608847, longitude 150.747016). Cultures were maintained on propagated lucerne (*M. sativa* L.) plants (Sequel cultivar) in each of the four CO_2_ and temperature combinations (conditions below) for at least six generations (c. 7 weeks) prior to the experiment. For the experiment, *M. sativa* (Sequel) were grown from seed (Heritage Seeds Pty, Adelaide, SA, Australia) in glasshouse rooms receiving supplemental light (15:9 light:dark) under the same conditions. Plants were grown in 70 mm diameter pots containing c. 700g of soil excavated from the Hawkesbury campus of Western Sydney University (location as above). The soil is typified as low-fertility sandy loam in the Clarendon Formation (Chromosol) (Barton et al., 2010), which has low bioavailable Si content of 10–17 mg kg^-1^ (Johnson et al., 2017).

### Growth Conditions and Experimental Procedures

Eighty lucerne plants were grown in each of four CO_2_ and temperature-controlled glasshouse chambers (320 plants in total) using a fully factorial design of ambient CO_2_ (aCO_2_; 400 μmol mol^-1^) and eCO_2_ (640 μmol mol^-1^) at ambient (aT) and elevated temperature (ambient + 4°C; eT). aT was set at 26/18°C day/night representing the average daily temperature (November to May) over the past 30 years for Richmond, NSW, Australia (Australian Bureau of Meteorology). eT (30/22°C day/ night) replicated the maximum predicted temperature increase for this region within this century (CSIRO, 2007–2016). Environmental conditions were monitored continuously throughout the experiment and temperature readings were verified with portable temperature loggers. To minimize ‘chamber effects’ associated with using four chambers, plants were circulated within each chamber every 5 days (apart from when plants were inoculated with aphids to avoid dislodgement of the insects) and chambers were swapped every c. 10 days by transferring plants between chambers and adjusting the environmental conditions accordingly. While this does not eliminate pseudoreplication, using this approach in these chambers has provided matching empirical results to fully replicated experiments, whether using multiple chamber replicates or multiple experimental runs (Johnson et al., 2016b).

Plants were irrigated with c. 70 ml of tap water (Si 3 ppm) three times a week. After growing for a further 2 weeks, half (40) of the plants continued to receive tap water (Si- plants or Si- soil hereafter) at the same intervals while the other half (selected at random) received 70 ml of 500 mg l^-1^ soluble silica in the form of NaSiO_3_.9H_2_O three times a week (Si+ plants or Si+ soil hereafter). When plants were 6 weeks old, 20 of the plants receiving the Si supplementation and 20 of the plants receiving tap water (selected at random) were inoculated with two teneral adult *A. pisum.* White mesh (organza) bags (125 mm × 170 mm) were applied tightly around the rim of all pots confining aphids to their allocated plants. After 2 weeks, bags were removed aphids counted (including colonization success; at least one aphid being present). Plants were cleaned free of soil with water and the number of active (pink) root nodules quantified. Maximum rooting depth was also quantified to provide a rudimentary measure of nodule density in order to give an indication as whether changes in nodule abundance were a function of root growth or nodule density on the roots (i.e., nodules per unit of root growth). Plants were freeze dried for 48 h and weighed. Leaves were separated from the stems and ball-milled to a fine powder prior to analysis for Si concentrations.

### Foliar Si Analysis

It was necessary to pool foliar samples (2–3 plants per sample), giving nine replicates of each treatment combination (CO_2_, temperature, Si application and aphid inoculation). Foliar Si concentrations were analyzed with X-ray fluorescence spectrometry using the method described by Reidinger et al. (2012). In summary, plant material was ground to a fine powder and pressed into 13 mm-diameter pellets. Foliar Si concentration was determined using a Niton XL3t XRF analyzer (Thermo Fisher Scientific, Inc., Waltham, MA, United States), for a measurement time of 30 s. Results we expressed as foliar Si concentration (as % of dry mass), calibrated against plant-certified reference material of known Si content (Garbuzov et al., 2011).

### Statistical Analysis

Goodness-of-fit tests, using the ‘goodfit’ function in the vcd package (Friendly, 2000), were employed to determine which distributions best described the data. Plant dry mass and nodule density were transformed (logarithm and square-root, respectively) prior to analysis to meet model assumptions and give residual diagnostic plots which fitted a normal distribution and showed least heteroscedasticity. Plant dry mass and nodule density were analyzed using analysis of variance with CO_2_, temperature, aphid presence, and Si supplementation included as fixed effects individually and in interaction with one another. Root nodule counts and aphid abundance were analyzed with generalized linear models with negative binomial error structures and log-link function using the same configuration of fixed effects as above. Aphid colonization success was analyzed in the same way but with binomial error structure and logit link function. Statistical tests of plant mass and nodulation were conducted on data collectively, before repeating the tests separately for Si- and Si+ plants since there were significant interactions between Si treatment and environmental treatments. Where non-significant effects were observed in full models (i.e., all factors included), non-significant factors were removed to determine whether this affected model inferences with more parsimonious models (e.g., fewer multi-way interaction terms were included in the model) – see Supplementary Table S1. All analysis was conducted in the R statistical package.

## Results

**FIGURE 1:**
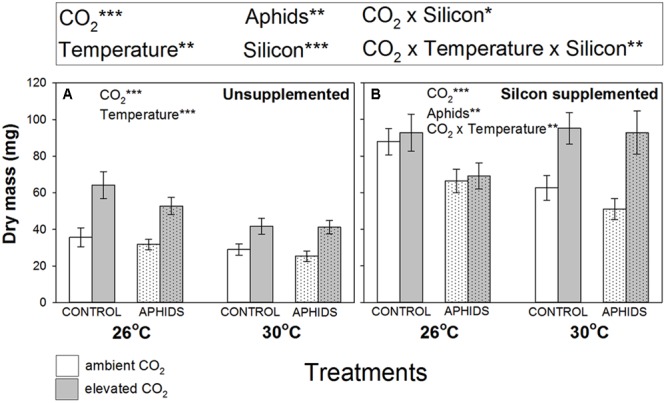
Impacts of CO_2_, temperature and aphid presence on dry mass of *Medicago sativa* when growing in **(A)** non-supplemented and **(B)** Si supplemented soil. Mean values ± standard error shown (*N* = 20) with statistically significant effects indicated ^∗^*P* < 0.05, ^∗∗^*P* < 0.01, and ^∗∗∗^*P* < 0.001. Significant factors for the whole experiment given in the upper panel (see **Table 1** for full results) and for Si– and Si+ plants separately in the respective graphical panels.

Plant growth was stimulated by eCO_2_ and Si supplementation by 41 and 93%, respectively (**Figure 1** and **Table 1**). In contrast, eT and aphid presence depressed plant growth by 13 and 17%, respectively (**Figure 1** and **Table 1**). Temperature depressed plant growth in Si- soil (**Figure 1A**), but not in Si+ soil (**Figure 1B**), though there was an interactive effect of CO_2_ and temperature in the latter, with eCO_2_ promoting plant growth more at eT than under aT conditions (**Figure 1B**).

**Table 1:** Results of statistical tests examining the effects of CO_2_, temperature (Temp), aphid presence and Si supplementation (Si) on plant growth, root nodulation, and foliar Si concentrations.

Plant response Model fixed effect	Dry mass^1^		Root nodules		Si concentration^1^	
	*F*_1,304_	*P*	RD_1,304_	*P*	*F*_1,94_	*P*
CO_2_	**51.86**	**<0.001**	**409.15**	**<0.001**	1.32	0.25
Temp	**10.28**	**<0.001**	**384.58**	**<0.001**	0.37	0.54
Aphids	**7.36**	**0.01**	384.57	0.90	1.17	0.28
Si	**150.55**	**<0.001**	**352.27**	**<0.001**	**15.22**	**<0.001**
CO_2_ × Temp	3.19	0.07	351.81	0.50	2.09	0.15
CO_2_ × Aphids	0.11	0.74	349.06	0.10	0.01	0.99
Temp × Aphids	0.63	0.43	345.60	0.06	1.03	0.31
CO_2_ × Si	**4.06**	**0.04**	344.47	0.29	0.07	0.80
Temp × Si	2.00	0.16	**336.69**	**0.01**	0.01	0.92
Aphids × Si	1.83	0.18	336.66	0.86	0.15	0.70
CO_2_ × Temp × Aphids	1.07	0.30	335.82	0.36	0.42	0.52
CO_2_ × Temp × Si	**8.70**	**<0.001**	334.95	0.35	0.43	0.51
CO_2_ × Aphids × Si	0.26	0.61	333.17	0.18	0.51	0.48
Temp × Aphids × Si	0.25	0.61	332.97	0.65	0.06	0.81
CO_2_ × Temp × Aphids × Si	0.06	0.81	332.95	0.90	0.84	0.36

*Statistically significant (*P* < 0.05) factors indicated in bold with Fisher’s (F) or residual deviation (RD) given depending on the models used. Analysis conducted on transformed data as indicated.*

*^*1*^Log transformed. Degrees of freedom in each column apply to all effects.*

**FIGURE 2:**
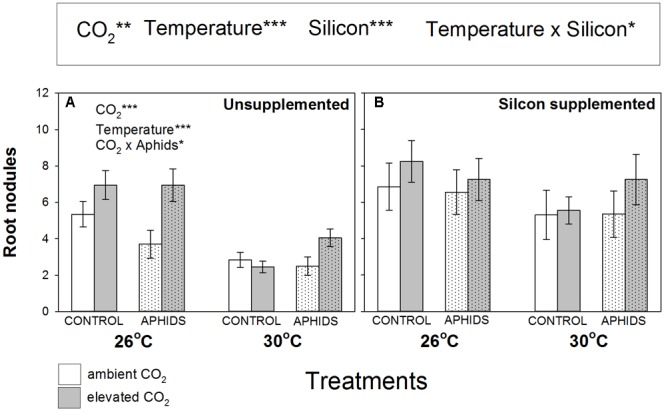
Impacts of CO_2_, temperature and aphid presence on root nodulation (number per plant) of *M. sativa* when growing in **(A)** non-supplemented and **(B)** Si supplemented soil. Mean values ± standard error shown (*N* = 20) with statistically significant effects indicated ^∗^*P* < 0.05, ^∗∗^*P* < 0.01, and ^∗∗∗^*P* < 0.001. Significant factors shown as per **Figure 1** legend.

Root nodulation increased when plants grew under eCO_2_ (+27%) and Si+ conditions (+50%) (**Figure 2** and **Table 1**), but eT caused significant declines in nodulation (-32%). In Si- soil, root nodulation patterns generally mirrored changes in plant growth (**Figures 1A, 2A**, respectively). Levels of root nodulation were universally high in plants growing in Si+ soil and other factors (CO_2_, temperature, and aphid presence) no longer had significant impacts (**Figure 2B**). This was particularly true for the negative impacts of eT, which was reversed under Si+ conditions, reflected by the significant interaction of these treatments (**Figure 2** and **Table 1**). Our rudimentary estimate of nodule density (nodules per unit of root depth) suggested this was not affected by CO_2_ (other than the weak interaction described below) but declined by 25% under eT (Supplementary Figure S1 and Supplementary Table S1). Nodule density increased (c. +45%) under Si+ conditions and, like nodule abundance, there was a significant interaction between Si treatment and temperature, whereby negative effects of eT were reversed under Si+ conditions (Supplementary Figure S1 and Supplementary Table S1). There was a very weak interaction between Si, aphids, and CO_2_.

**FIGURE 3:**
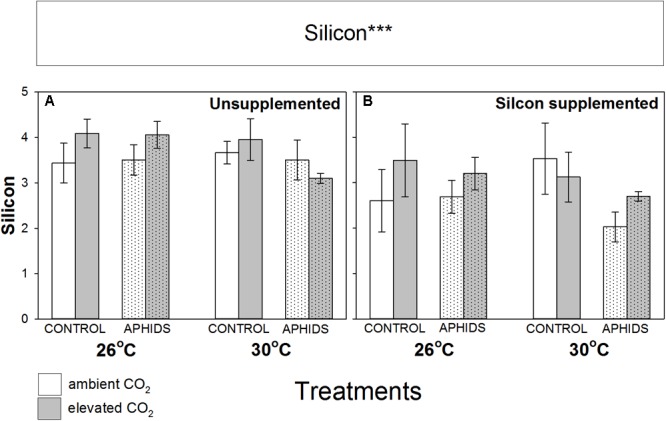
Impacts of CO_2_, temperature and aphid presence on Si concentrations of *M. sativa* foliage (% dry mass) when growing in **(A)** non-supplemented and **(B)** Si supplemented soil. Mean values ± standard error shown (*N* = 9) with statistically significant effects indicated ^∗∗∗^*P* < 0.001. Significant factors shown as per **Figure 1** legend.

Si concentration in the foliage was unaffected by CO_2_, temperature and aphid presence, though unexpectedly there was a small but significant decline in foliar Si concentrations when growing in Si+ soil (**Figure 3** and **Table 1**).

**FIGURE 4:**
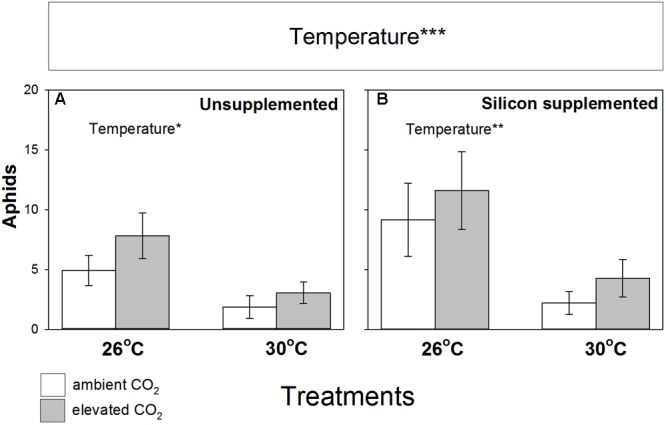
Impacts of CO_2_ and temperature on aphid (*Acyrthosiphon pisum*) abundance (number per plant) when feeding on *M. sativa* plants growing in **(A)** non-supplemented and **(B)** Si supplemented soil. Mean values ± standard error shown (*N* = 20) with statistically significant effects indicated ^∗∗∗^*P* < 0.001. Significant factors shown as per **Figure 1** legend.

Aphid abundance was not significantly affected by eCO_2_ (**Figure 4**), although colonization success increased by 14% under eCO_2_ (**Table 2**). In contrast, eT caused substantial declines (-65%) in aphid abundance and reduced their ability to colonize plants, falling by 48 and 43% on Si- and Si+ plants, respectively (**Table 2**). Aphid populations at eT were similar regardless of Si treatments. In short, aphid abundance was always lowest at 30°C and Si promotion of plant growth and nodulation was decoupled from aphid performance, such that Si+ conditions led to increased nodulation (and potentially BNF) without increasing aphid numbers.

**Table 2:** Results of statistical tests examining the effects of CO_2_, temperature and Si supplementation on aphid abundance and colonization success.

Plant response Model fixed effect	Aphid abundance		Aphid colonization	
	RD_1,159_	*P*	RD_1,159_	*P*
CO_2_	177.62	0.16	**215.23**	**0.026**
Temp	**162.39**	**<0.001**	**197.21**	**<0.001**
Si	160.42	0.16	196.16	0.30
CO_2_ × Temp	160.24	0.68	195.90	0.61
CO_2_ × Si	160.24	0.93	195.90	0.99
Temp × Si	160.03	0.65	105.89	0.95
CO_2_ × Temp × Si	159.91	0.73	194.50	0.24

*Statistically significant (*P* < 0.05) indicated in bold with residual deviation (RD) given.*

**FIGURE 5:**
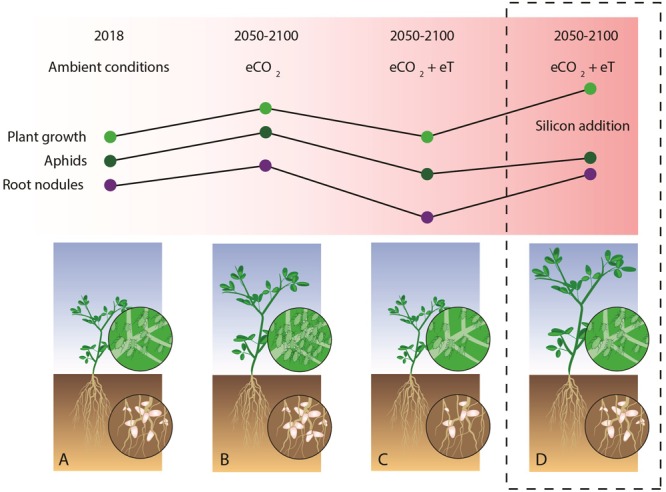
Graphical summary showing how Si supplementation affects *M. sativa* growth, root nodulation and susceptibility to *A. pisum* in current and predicted changes to the climate and atmosphere. Compared with ambient CO_2_ and temperature conditions **(A)**, eCO_2_ had beneficial effects on plant growth and nodulation **(B)**, but these were negated when acting in combination with predicted increases in temperature **(C)**. Si supplementation **(D)** restored root nodulation to comparable levels as those seen under eCO_2_ conditions (without warming) and stimulated plant growth beyond this. Aphid responses were decoupled from changes in nodulation and growth with Si supplementation.

The key findings of this study are summarized in **Figure 5** which held true when non-significant terms were dropped from models for parsimony (see Supplementary Table S2). **Figures 5A–C** shows how aphid abundance mirrors patterns of nodulation and plant growth in non-supplemented soils, but this becomes decoupled in Si+ soils, where Si supplementation restores the fertilizing effects of eCO_2_ on *M. sativa* at higher temperatures without affecting aphid populations.

## Discussion

Results from this study suggest that Si supplementation may mitigate the negative impacts of eT on plant growth in *M. sativa* which was potentially due to stimulation of root nodulation, despite the reduction in nodulation at higher temperatures reported in previous studies (e.g., Ryalls et al., 2013b). Even more advantageously, this increased nodulation did not increase susceptibility to an aphid pest at eT, which had previously been observed for Si-induced increases in nodulation at ambient temperatures (Johnson et al., 2017).

Aphid abundance was strongly suppressed by eT and this most likely explains why aphids did not benefit from increases in plant growth and nodulation that arose under Si+ conditions under eT. While aphid numbers often increase with higher temperatures via faster development and increased fecundity, this increase ceases abruptly over a certain temperature threshold because of the adverse effects on, for example, embryo development and maturation (Ryalls and Harrington, 2017). This temperature threshold depends on species, aphid biotype, and geographical region (Awmack and Leather, 2007). *A. pisum* has adapted to the warmer climate of Australia since introduction in the 1970s (Ryalls et al., 2013a). Some populations are able to function at temperatures above 35°C, although their optimum temperature is said to be c. 20–25°C (Ryalls, 2016) and temperatures above 28°C are likely to reduce aphid growth and development (Bieri et al., 1983; Lamb and MacKay, 1988; Mackay et al., 1993). Aphid biotypes with certain secondary bacterial endosymbionts may cope better with higher temperatures, however, since there have been several reports of endosymbionts alleviating the effects of heat stress (Montllor et al., 2002; Russell and Moran, 2005; Dunbar et al., 2007). To our knowledge, studies have not yet addressed how bacterial endosymbionts might change in response to eCO_2_ *and* eT but endosymbionts could partially facilitate adaptation to climate and atmospheric change (Sun et al., 2016; Ryalls and Harrington, 2017).

Several studies using temperature gradient greenhouses have examined the impacts of eCO_2_ and eT on legume performance, including root nodulation (Aranjuelo et al., 2006, 2008; Erice et al., 2006, 2007). These studies report a general trend for eCO_2_ promoting nodulation, but only at the elevated experimental temperatures. This was probably because the elevated temperature range used in experiments (c. 24°C; Aranjuelo et al., 2008) was still within the optimal range (19–25°C) for nodulation in temperate legumes, so inhibitory effects of temperature on nodulation wouldn’t necessarily have occurred (Aranjuelo et al., 2014). When temperature was elevated beyond 25–30°C, root nodulation in *M. sativa* has been reported to decrease by 22% under ambient CO_2_ (aCO_2_) and by 56% under eCO_2_ (Ryalls et al., 2013b).

Despite increasing evidence that the effects of eCO_2_ are often modified by eT, and *vice versa*, comparatively few studies manipulate both factors in tandem (Robinson et al., 2012). In the present study we established that positive impacts of eCO_2_ on plant traits were not seen to the same extent when eT conditions were applied. This study therefore lends support to the notion that, wherever feasible, multiple environmental factors should be tested (Newman et al., 2011; Lindroth and Raffa, 2016). Crucially, Si supplementation had consistently stronger impacts on plant traits across a range of environmental conditions and regardless of whether plants were challenged by herbivores.

A counterintuitive finding of the study was that Si supplementation actually reduced concentrations of Si in the foliage. Si may have promoted plant growth to such an extent that Si became ‘diluted’ in foliage, or else had not had time to accumulate in plant tissues over the duration of the study. A similar trend in foliar Si was previously observed in this system however, associated with rapid plant growth, increases in root nodulation and synthesis of amino acids (Johnson et al., 2017). In addition to any increased nutritional value, the lower concentrations of Si in foliage of Si+ plants may explain why Si supplementation did not increase plant resistance to aphids.

Our results demonstrate conclusively the benefits of Si supplementation for root nodulation: root nodule abundance was always increased in plants growing in Si+ soil and other factors, whether CO_2_, temperature and aphid presence no longer had significant impacts on nodule abundance. The mechanisms by which Si is so effective at promoting nodulation are not well-understood, but could include changes in soil conditions, increased root growth (and potential invasion sites), higher abundance of bacteroids and symbiosomes, together with the synthesis of compounds that upregulate nodulation genes (as discussed by Johnson et al., 2017). The increased nodule density reported in the present study tentatively suggests that greater nodule abundance was not merely a function of increased root growth. Further work is needed, but Si could provide a useful tool for mitigating some of the negative impacts of climate change on crop production – in this instance maintaining nodulation rates of *M. sativa* in warmer climates. Moreover, other studies suggest Si could redress negative effects of eCO_2_ on plant–herbivore interactions. For example, herbivore damage to roots of sugarcane was exacerbated under eCO_2_ conditions, but application of Si reversed these effects and stimulated crop growth (Frew et al., 2017). Intervention strategies could include targeted application of Si (e.g., furnace slag), selection of plant lines that naturally take up large amounts of Si (McLarnon et al., 2017) and remediation of soils deficient in bioavailable Si (silicic acid) (Guntzer et al., 2012; Johnson et al., 2016a).

## Author Contributions

SJ, JR, AF, and AG: conceived the experimental design; SJ, JR, AF, and AG: acquired and processed data with SH undertaking Si analysis; JR: analyzed the data and all authors contributed to the interpretation and drafting of the manuscript.

## Conflict of Interest Statement

The authors declare that the research was conducted in the absence of any commercial or financial relationships that could be construed as a potential conflict of interest.
