# Supplementary material for: Benefits from Below: Silicon Supplementation Maintains Legume Productivity under Predicted Climate Change Scenarios
Source: Front Plant Sci. 2018 Feb 20;9:202. doi: 10.3389/fpls.2018.00202 (PMC5829608; doi:10.3389/fpls.2018.00202)
Supplement: Supplementary file 1 [file Table_1.docx]

**Supplementary Material**

**Fig. S1.** Impacts of CO_2_, temperature and aphid presence (bars with dots) on root nodulation density (nodules per cm of root depth) of *M. sativa* when growing in (A) non-supplemented and (B) Si supplemented soil. Mean values ± standard error shown (N = 20) with statistically significant effects indicated **P* < 0.05, ***P* < 0.01 and ****P* < 0.001 (see Table 1 for results of statistical analysis).

**Table S1.** Results of the statistical test examining the effects of CO_2_, temperature (Temp), aphid presence and Si supplementation (Si) on root nodule density. Statistically significant (*P* <0.05) terms indicated in **bold**.

| Plant response  Model fixed effect | Root nodule density^1^ | |
| --- | --- | --- |
|  | F_1,304_ | *P* |
| CO_2_ | 0.27 | 0.60 |
| Temp | **10.35** | **<0.001** |
| Aphids | 0.01 | 0.94 |
| Si | **17.93** | **<0.001** |
| CO_2_ 🞨 Temp | 1.64 | 0.20 |
| CO_2_ 🞨 Aphids | 2.61 | 0.11 |
| Temp 🞨 Aphids | 1.19 | 0.28 |
| CO_2_ 🞨 Si | 2.45 | 0.12 |
| Temp 🞨 Si | **7.35** | **0.01** |
| Aphids 🞨 Si | 0.33 | 0.57 |
| CO_2_ 🞨 Temp 🞨 Aphids | 0.07 | 0.79 |
| CO_2_ 🞨 Temp 🞨 Si | 1.42 | 0.23 |
| CO_2_ 🞨 Aphids 🞨 Si | **4.46** | **0.04** |
| Temp 🞨 Aphids 🞨 Si | 0.17 | 0.68 |
| CO_2_ 🞨 Temp 🞨 Aphids 🞨 Si | 0.26 | 0.61 |

^1^Square-root transformed

**Table S2**. Results of models from Table 1 dropping non-significant factors. Details as described for Table 1 which provide results for all factors and interactions between factors.

| Plant response  Model fixed effect | Root Nodules^1^ | | Root nodule density^2^ | | Si Concentration^3^ | |
| --- | --- | --- | --- | --- | --- | --- |
|  | RD_1,311_ | *P* | F_1,316_ | *P* | F_1,108_ | P |
| CO_2_ | **400.01** | **0.002** |  |  |  |  |
| Temp | **376.06** | **<0.001** | **11.20** | **<0.001** |  |  |
| Si | **344.47** | **<0.001** | **19.04** | **<0.001** | **16.13** | **<0.001** |
| CO_2_ 🞨 Temp | 344.03 | 0.51 |  |  |  |  |
| CO_2_ 🞨 Si | 344.47 | 0.31 |  |  |  |  |
| Temp 🞨 Si | **336.69** | **0.01** | **6.66** | **0.01** |  |  |
| CO_2_ 🞨 Temp 🞨 Si | 334.57 | 0.39 |  |  |  |  |

^1^Excluded non-significant factors: Aphids

^2^Excluded non-significant factors: Aphids, CO_2_

^3^Excluded non-significant factors: Aphids, CO_2_, Temp
